# An Initial Look at Federal Offices of Research and Technology Applications

**DOI:** 10.6028/jres.125.033

**Published:** 2020-11-19

**Authors:** Nicole Gingrich, Michael Hall, Isaac Patterson

**Affiliations:** 1 National Institute of Standards and Technology, Gaithersburg, MD 20899, USA

**Keywords:** federal laboratory, Office of Research and Technology Applications, technology transfer, technology transfer office

## Abstract

In *Science—The Endless Frontier*, Vannevar Bush wrote that reaping the potential benefits of science conducted at federal laboratories requires the discoveries made in the laboratories be transferred to society. In federal laboratories, Offices of Research and Technology Applications (ORTAs) are tasked with transferring laboratory-developed technologies to the market, allowing society to reap the benefits provided by scientific investments. In fiscal year 2016, the Technology Partnerships Office of the National Institute of Standards and Technology (NIST) conducted a first-of-its-kind survey of the ORTAs of more than 50 federal laboratories to obtain information on their organization and operation. We present descriptive analyses of the responses to this survey in two topical areas: organizational characteristics and technology transfer characteristics. We disaggregated the data across the dimension of budget size to describe similarities and differences in responses across the budget categories. Among the relationships we observed, we found that ORTAs with larger technology transfer budgets report higher frequencies of conducting internal technology transfer activities, such as patent prosecution (e.g., drafting patents, filing patent applications, and responding to actions from the patent office) and market analysis. Additionally, we provide context to the data by summarizing the relevant research on ORTAs at universities, and we present potential inferences that may be drawn from that body of research and applied to the data on ORTAs at federal laboratories.

“Technology transfer is the process by which existing knowledge, facilities, or capabilities developed under federal research and development funding are utilized to fulfill public and private needs.”

—The Federal Laboratory Consortium [1]

## 1 Introduction

In 2016, the Technology Partnerships Office (TPO) of the National Institute of Standards and Technology (NIST) conducted a survey of Offices of Research and Technology Applications (ORTAs)^1^1 ORTAs operate under various names, such as Technology Transfer Offices (TTOs) or Offices of Technology Transactions (OTTs). For consistency, we will refer to any group within an organization for which the primary role is the external transfer of technology as an ORTA. at federal laboratories and agencies. This work describes the responses to that survey and places this information in the context of existing research on ORTAs at universities and federal laboratories.

This paper is an extension of the description of survey responses previously reported in a Technology Transfer Brief [2]. Herein, we provide additional insight by disaggregating the available data by size of budget for respondent ORTAs. We undertake two tasks in this paper: first, to describe the survey we conducted and disaggregate the responses to the survey across budgetary categories, and second, to review the extant literature on ORTAs with an emphasis on research that provides context to the significance of the organizational characteristics described by the survey results. The benefit of undertaking these tasks was articulated by Lafuente and Berbegal-Mirabent [3] when they wrote, “organization practices and resource allocation strategies explain a significant proportion of the variation of [ORTA] performance.” We caution, however, that while we review literature on ORTAs, many of those studies have conducted complex statistical analyses (e.g., applying regression and forecasting models), whereas we have not. This paper reviews the current state of ORTAs and summarizes the results of a survey of ORTAs at governmental laboratories.

This work also serves to increase the information on federal ORTAs available to researchers studying federal technology transfer. The survey described herein is, to some extent, a federal analog of surveys on ORTAs conducted by the Association of University Technology Managers (AUTM) [4]. Technology transfer researchers have used these data for decades to examine how characteristics of university ORTAs influence the efficiency and efficacy of technology transfer. There is a much smaller body of research on federal technology transfer; the reason for this was suggested by Link *et al.* [5] and Link and Oliver [6] to be limited availability of federal data.

This work focused on patents, invention licenses, and cooperative agreements. These are not the only mechanisms for technology transfer and are not the goal of every research effort at a federal laboratory. Hourihan [7] listed many potential technology transfer mechanisms, including tacit knowledge, “peer reviewed scientific publications, conference papers, reference manuals, conferences, presentations, and other ‘grey’ literature as well as through joint ventures or contracts.” Although these mechanisms are useful means for technology transfer, and many of these mechanisms are used by federal laboratories, we focused on patents, invention licenses, and cooperative agreements because they represent much of the work of federal ORTAs.

The remainder of this paper is organized as follows. Section 2 provides institutional background and history on federal ORTAs. Section 3 describes the survey methodology. Section 4 provides a description of survey results and places the results within the context of academic literature on university ORTAs, where applicable. Section 5 provides concluding remarks.

## 2 Institutional Background and History of Federal ORTAs

As the director of the Office of Scientific Research and Development during World War II, Dr. Vannevar Bush presented perhaps the earliest conceptualization of the benefits of commercializing the results of federal investments in science and technology. In *Science—The Endless Frontier* [8], Bush wrote that science has the potential to provide an expansive array of benefits to society. However, to obtain these benefits “the flow of new scientific knowledge must both be continuous and substantial.”

The Stevenson-Wydler Act of 1980 (PL 96-480) [9] requires federal laboratories to establish ORTAs and engage in technology transfer. The Stevenson-Wydler Act defines a federal laboratory as “any laboratory, any federally funded research and development center (FFRDC), or any center that is owned, leased, or otherwise used by a Federal agency and funded by the Federal Government, whether operated by the Government or by a contractor.”^2^2 The Federal Laboratory Consortium [10] regularly publishes *The Green Book*, a handbook designed to overview major federal technology transfer policy and legislation. It provides a more thorough review of the body of laws pertaining to federal technology transfer policy than we could succinctly provide here.

The statute defines the minimally required, specific functions of ORTAs at federal laboratories. ORTAs must:

• prepare assessments of selected research and development (R&D) projects and technologies in the laboratory that may have potential commercial applications;
• provide and disseminate information to state and local governments and private industry about potentially applicable federally owned or originated technologies, products, processes, and services;
• cooperate with and assist the Federal Laboratory Consortium (FLC), the National Technical Information Service (NTIS), and other organizations that link the R&D resources of the federal government to potential users in state and local governments and private industry; and
• provide technical assistance to state and local government officials and participate in regional, state, and local programs designed to facilitate or stimulate the transfer of technology for the benefit of the region, state, or local jurisdiction in which the federal laboratory is located [9].

Although these functions are clearly defined in the act, there are other important tasks commonly performed through ORTAs that are infrequently studied. These include negotiating licenses on intellectual property, coordinating collaborative agreements, marketing patent portfolios and available resources (e.g., user facilities), and educating stakeholders about their existence and purpose both inside and outside of the laboratory.

The exact number of federal laboratories across the federal government is not well documented. While referring specifically to high-containment laboratories, Dr. Nancy Kingsbury, then managing director of applied research and methods of the Government Accountability Office, testified before the House Committee on Energy and Commerce [11] and stated, “[N]obody knows how many there are.” This sentiment is likely applicable to the broader body of federal laboratories. One may roughly approximate the magnitude of the count of federal laboratories from the FLC [12], which states that it has over 300 federal laboratories as members.

In 1987, Executive Order 12591 [13], Facilitating Access to Science and Technology, established the Interagency Workgroup on Technology Transfer (IAWGTT). The executive order states that the director of the Office of Science and Technology Policy shall “convene an interagency task force comprised of the heads of representative agencies and the directors of representative Federal laboratories, or their designees, to identify and disseminate creative approaches to technology transfer from Federal laboratories.”

Anecdotally, ORTAs have reached out to other ORTAs independently to inquire about their structure and processes for the purpose of learning from others, but this information was not previously aggregated or made available. Through discussions at IAWGTT meetings, it became apparent that formal data collection and sharing would be useful both within IAWGTT and among the broader audience of researchers studying federal technology transfer.^3^3 Agencies agreed to participate in the study based on the agreement that specific agencies would not be identified in publications resulting from the survey. Due to this agreement, we do not identify specific ORTAs in this work. This interest, generated during IAWGTT discussions, was the impetus for the survey presented in this work. NIST, as the host agency of the IAWGTT and FLC, and the producer of the annual Federal Technology Transfer report, volunteered its staff to collect data for the purpose of building knowledge of how ORTAs are structured and how they operate.

In fiscal year 2016, the U.S. government spent a combined $43 billion on intramural research and research at FFRDCs [14]. Much of this research is conducted at federal laboratories. Decades of federal policies have incentivized the federal government’s transfer of the technologies resulting from federally funded research to the market for commercial use (i.e., from laboratory to market). ORTAs play a critical role in this laboratory-to-market process.

## 3 Survey Methodology and Structure

In fiscal year 2016, NIST administered a project through the IAWGTT to collect cross-sectional data on federal ORTAs. Gingrich [2] presented an initial compilation of the 63 survey responses. This work extends that earlier research by disaggregating the descriptive analysis across the dimension of laboratory size.

The survey^4^4 The survey contained 32 questions organized into seven parts. These parts were titled: Structure, Personnel, Patenting, Agreements, Marketing, Research, and Recognition. Each subsection contained some combination of questions in multiple choice, numerical response, and open-ended formats. The full text of the survey can be found here: https://www.nist.gov/system/files/documents/2018/05/22/orta_tto_questionnaire_final.pdf created by the NIST TPO collected quantitative and qualitative data regarding federal government ORTAs during fiscal year 2016. The 11 agencies within the IAWGTT participated in two ways: first, to review and approve the survey instrument’s questions; and second, to respond to the survey regarding their own ORTAs. A two-step process involved the lead agency representatives in the IAWGTT filling out the survey, followed by additional ORTAs within agencies, as identified by lead agency representatives.^5^5 This technique of surveying, known as “snowball sampling” or “respondent-driven sampling,” was appropriate for this study because the heads of representative agencies within the IAWGTT generally knew the number of ORTAs within their organizations, and they were able to provide appropriate contacts. In total, the federal laboratories and agencies returned 63 ORTA-level surveys to NIST.^6^6 NIST received at least one response from all 11 federal agencies. We conditioned our analysis below on a completed response to questions regarding the ORTA budget. This condition resulted in a final sample size of 58 ORTAs.

Within the survey, TPO asked questions regarding “traditional technology transfer.” Traditional technology transfer operations include activities involved in patenting, license negotiations, and collaborative research agreements. This distinction is important for an accurate interpretation of budget and employment numbers.

We organized the survey questions into two categories: organizational characteristics and technology transfer characteristics. Academic literature on technology transfer—federal and university—has shown that the effectiveness and efficiency of ORTAs are related to many of these characteristics. Heisey and Adelman [15] articulated this point when they wrote, “In our analysis of 192 universities over the 13-year period 1991–2003, variables related to technology transfer office (TTO) characteristics most consistently explained variation in university licensing revenue.”

The following list describes each characteristics category and the related information collected.

• **Organization Characteristics—**The survey asked respondents a series of questions about their organization, including age, laboratory structure, budget size, and budget source. We excluded budget size from this description because we disaggregated the population of responses along that dimension.
  o **Age—**The age data describe the number of years the ORTA has functioned within the specific level of the agency; it is independent of geographic or organizational location of the ORTA itself.
  o **Laboratory Structure—**The organizational structure data describe the structure of the laboratory that the ORTA supported, chosen from the following options: government-owned, government-operated (GOGO), government-owned, contractor-operated (GOCO), and a combination of GOGO and GOCO [17]. The FFRDCs were captured within the GOGO/GOCO combination response.^7^7 Federal laboratories operated by the government and laboratories operated by contractors conduct technology transfer differently due to difference in the laws that govern the technology transfer activities of each. This difference arises from the employment status of researchers at these laboratories. Employees at GOGOs may be federal employees, who are governed by the Stevenson-Wydler Act. Employees at GOCOs, conversely, are employees of the contractor (often a university) and thus are not federal employees, and they are therefore governed by the Bayh-Dole Act (PL 96-517) [16]. A description of the types of laboratories was provided by Snyder and Thomas [17].FFRDCs are a subset of GOCOs that are designed to meet the nation’s long-term needs in specific areas of research and development. FFRDCs are assigned authority under the Federal Acquisition Regulation (FAR), which enables them to “adapt quickly to provide new and cutting-edge scientific support and technical expertise” [16].
  o **Budget Source—**The budget source data describe the source from which the ORTA received its funds to carry out traditional technology transfer functions. These sources include, but are not limited to, licensing royalties and directly appropriated funds.
  o **Total Full-Time-Equivalent (FTE)—**The employment data describe number of federal FTE and nonfederal FTE employees within each ORTA who were allocated specifically to traditional technology transfer activities.
• **Technology Transfer Characteristics—**These characteristics describe three areas of technology-transfer activities: patenting, licensing, and cooperative agreements.
  o **Patent Prosecution Funding Source—**These data describe the source of funds used by the ORTA to prosecute patents.^8^8 Patent prosecution refers to drafting and filing patent applications and responding to requests from the patent office. ORTAs have different approaches to paying for the costs associated with patenting. Six categories were identified from ORTAs’ responses for the funder of patent prosecution: ORTA, laboratory, Office of General Counsel (OGC, or equivalent), a combination of either two or three categories, other, and not applicable. The category of not applicable simply means that the ORTA does not fund the patenting process; the ORTA may have more of a management role in patenting, rather than an active role.
  o **Patent Application Filing Source—**These data describe whether patent attorneys utilized by the ORTA were external or internal to the ORTA. The survey offered three categorical responses for the location of patent attorneys: internal to the ORTA, external counsel, or a combination of internal and external.
  o **Market Analysis—**The market analysis data describe whether the ORTA conducts market analysis internally or utilizes an outside entity to complete a formal analysis.
  o **Royalties to Inventors—**These data describe the style of distributing royalty payments to inventors of associated patents. When royalty payments are received by the ORTA from the licensee, part of the income is distributed to the inventor(s). While the National Technology Transfer and Advancement Act of 1995 [18] describes the minimum amount an ORTA must pay to inventors, ORTAs can go above and beyond those requirements.
  o **Remaining Royalty Receiver—**The remaining royalty receiver describes the group, organization, office, or person that receives royalty income once the inventor(s) has received their allotment.
  o **Collaborative Agreements—**These data describe the role the ORTA plays in the approval of collaborative research and development agreements (CRADAs). This survey question focused specifically on CRADAs. CRADAs may or may not be handled through the ORTA. In addition, the ORTA’s level of responsibility varies. The ORTA, or another organization within the specific laboratory, may use a tool other than a CRADA to support collaborations; however, this section looks specifically at CRADAs.

## 4 Summary of Survey Responses

In this section, we present the survey responses in a series of figures to depict survey results. For each survey response category, we discuss theoretical impacts that the variable may have on technology transfer outcomes (e.g., number or value of technology licenses). Subsequently, we describe the differences in survey responses across budget size categories, listed below. Finally, we review the extant literature on ORTAs at universities and governmental laboratories and summarize findings from this literature related to data collected from the survey. While our analysis is solely a summary of survey responses, other studies have employed statistical techniques such as regression analyses and forecasting models. We organized the responses from the ORTAs into groups by budget size. The three budget sizes are:

• less than $1 million,
• $1 million up to $3 million, and
• greater than $3 million.

These budget categories were chosen for theoretical and pragmatic reasons. First, we expected that the resources available to the ORTA likely influenced how the ORTA operates. Therefore, segmenting ORTAs into these categories allowed us to observe the characteristics of each group individually. The tripling of budget from the $1 million threshold to the $3 million threshold represents a significant increase in resources. Second, these threshold values were easy for respondents to understand and conceptualize. Third, these thresholds allowed for a categorization that may be used by others. An earlier summary of these survey results presented to the IAWGTT included additional budget categories; however, their inclusion did not substantially alter the overall observations.

We begin our summary of survey responses with an overview. In theory, ORTAs with more resources may have greater ability to process invention disclosures, patent applications, and license agreements within a given time period. However, it is not clear if the return to scale—the relationship between an increase in an input (e.g., funding) and the change in output (e.g., license agreements)—is increasing or decreasing. An increasing return to scale would mean that each additional dollar of funding led to greater increases in technology transfer outputs than the previous dollar; conversely, a decreasing return to scale would mean that an additional dollar of funding would lead to smaller increases in technology transfer outputs than the previous dollar.

**Fig. 1:**
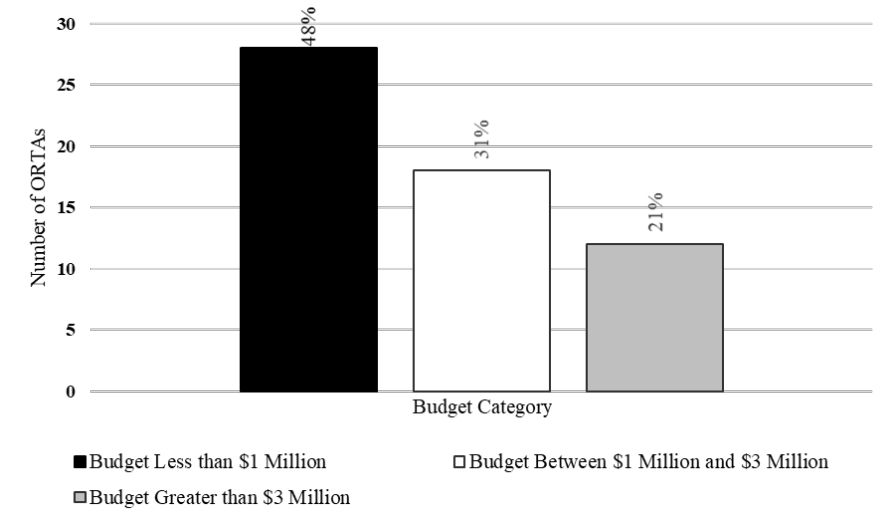
Number and proportion of ORTAs in each budget level (*n* = 58).

Figure 1 shows the number of ORTAs within each budget category. The most reported budget size was less than $1 million, which accounted for 28 of the ORTAs. Further, 18 ORTAs reported a budget size of $1 million to $3 million, 12 ORTAs had budgets of greater than $3 million, and 5 ORTAs did not disclose their budgets. The 5 ORTAs that declined to report budget levels were not considered in the descriptive analysis below. The final sample with complete data consisted of 58 ORTAs.

Comparison across budget levels provided a framework for further understanding trends in the data. We present these comparisons as a statement of empirical fact with limited speculation as to the nature or cause of these described relationships, and we make no inferences about causal relationships. It is plausible that the observed characteristics (e.g., funding levels, source of patent filing, number of employees) were determined by decisions made outside of the ORTA (e.g., laboratory administration). As such, observations about characteristics or their relationships herein should be considered descriptions of conditional correlations and not causal in nature.

Several studies have examined the relationship between university ORTA resources and technology transfer performance. In general, larger organizations have higher levels of technology transfer activities. However, these studies have reported conflicting results regarding ORTA resources and the efficiency and returns to scale of technology transfer activities. Siegel *et al*. [19] found constant returns to scale for the number of licenses and increasing returns to scale for licensing revenues. Conversely, Chapple *et al*. [20] found there were decreasing returns to scale at UK universities, and Foltz *et al*. [21] found “decreasing returns to scale in the technology transfer bureaucracy.” Swamidass and Vulasa [22] found that ORTAs that faced resource constraints (i.e., limited funds and personnel) chose to focus their efforts on patent prosecution over marketing innovations and pursuit of licenses.

We next consider the age of ORTAs. The age of an ORTA, measured as years since it was first established, may capture organizational experience and knowledge. It is possible that older ORTAs may embody more experience and thus are better able to identify valuable inventions or process agreements in a timely manner. Conversely, it is possible that younger ORTAs are more responsive to new processes and experience less organizational inertia.

The median ORTA age was greater than 20 years. This measure of age was consistent across all budget categories, with the median age for each budget category being greater than 20 years. This finding aligns with the fact that the Federal Technology Transfer Act of 1986 [23], which mandated the creation of ORTAs at federal laboratories, is more than 30 years old.

The literature suggests there is a positive relationship between ORTA age and technology transfer performance. Age of the university ORTA has been found to have a positive impact on patenting [24–25], licensing revenues [15, 24–25], and overall technology transfer performance [26]. The collective results of research on age suggest that older ORTAs at government laboratories are likely to correlate with higher technology transfer output.

We next describe the funding source of ORTAs. The source of funds for ORTA operations may incentivize—provide motivation to behave in a particular way or take particular actions—the decision makers to alter their approach to technology transfer. In theory, if an ORTA receives funds from a stable and predictable source, then it may be more patient with licensing negotiations. The reverse is also plausible: If an ORTA relies on licensing revenues to fund its operations, then it may be more aggressive in technology licensing practices and attempt to maximize licensing revenues.

**Fig. 2:**
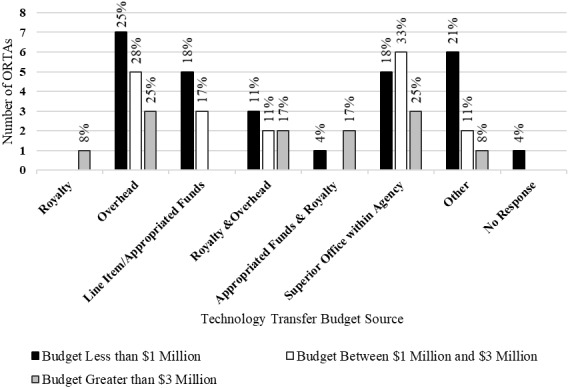
Sources of funding for ORTAs by budget category (*n* = 58).

Figure 2 shows the sources of funding for ORTAs by budget category. The sources of funding for ORTAs are diverse for all budget categories. For Fig. 2, and subsequent figures in this work, the height of a bar represents the count of ORTAs that gave a particular response. The percentage above a bar is the proportion of ORTAs in each budget category that gave that response. For the 58 responses, “overhead” was the most common response. The most common responses for individual budget categories were as follows: ORTAs with budgets of less than $1 million reported overhead as the most common funding source; ORTAs with budgets between $1 million and $3 million reported both overhead and a superior office within the agency as the most common sources; and ORTAs with budgets greater than $3 million reported a superior office within the agency as the most common source.

The ORTAs with budgets of less than $3 million reported lower rates of royalties as a funding source. Less than 15% of ORTAs with budgets less than $3 million reported that royalties from licenses were at least a portion of their funding sources. More than 40% of ORTAs with budgets greater than $3 million reported that royalties provided a portion of their budget.

Thursby *et al*. [27] surveyed university ORTAs on, among other things, their source of funding. They found 42% of budgetary support for university ORTAs came from a “line item” in the budget, while 43% of their budgetary support came from royalties and licensing fees. One may compare these figures to the reported budget sources for federal ORTAs and observe that royalties constitute a smaller proportion of budgetary support for federal ORTAs across all budget categories. We could find no prior work that examined the relationship between budget source and technology transfer performance.

We next discuss the employment levels reported by the ORTAs. Employment is a measure of the labor input into the technology transfer process. We expected ORTAs with more FTEs to have greater numbers of technology transfer outputs. The directionality—whether more FTEs lead to greater output or greater output leads to additional hiring—is unclear. Additionally, like budget size, whether return to scale of labor inputs are increasing or decreasing is not clear and may in fact be different than returns to scale for monetary inputs.

**Fig. 3:**
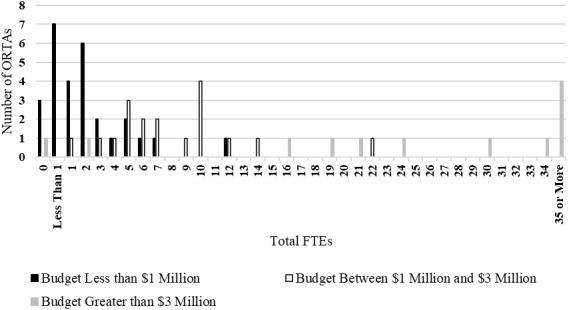
Total FTEs by budget category (*n* = 58).

Figure 3 shows the number of total FTEs reported by ORTAs. These figures represent the combined total of federal and nonfederal FTEs allocated to traditional technology transfer activities. Median values for budget categories were as follows: ORTAs with a budget less than $1 million reported a median of 2 FTEs; ORTAs with a budget between $1 million and $3 million reported a median of 7 FTEs; and ORTAs with a budget of more than $3 million reported a median 27 FTEs. One may observe from the figure that ORTAs with larger operating budgets generally had more employees. This positive relationship is intuitive because ORTAs with larger budgets may have these resources for the purpose of paying larger numbers of staff.

Previous research has found that, in general, ORTA size (e.g., FTEs) is positively correlated to technology transfer output. Multiple studies have found a positive relationship between the size of the ORTA staff and licensing revenues [15, 25, 27]. Caldera and Debande [28] found that having more staff was positively related to additional licensing activity but not additional licensing revenues at Spanish universities. Foltz *et al*. [21] and Rogers *et al*. [26] found that a greater number of ORTA staff was positively correlated to levels of patent prosecution. Rogers *et al*. [26] also found that a greater number of ORTA staff was correlated with increased numbers of invention disclosures.

**Fig. 4:**
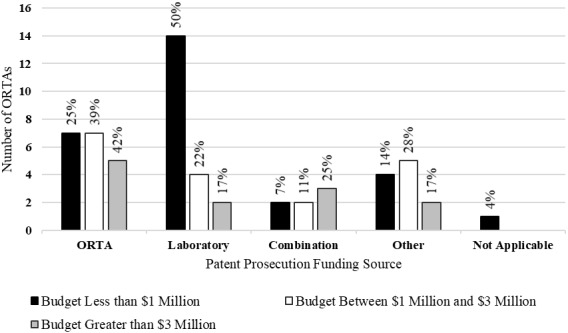
Patent prosecution funding source by budget category (*n* = 58).

We next discuss the source of funding for patent prosecution. To the best of our knowledge, there is no body of literature that examined the specific source of funds for patent prosecution and its effect on technology transfer efficacy or efficiency. Figure 4 shows the funding source of patent prosecution for ORTAs.

From Fig. 4, the most common funding source for all ORTAs was the corresponding laboratory. However, ORTAs with larger budgets funded patent prosecution from internal ORTA funds at a higher rate than ORTAs with smaller budgets. ORTAs with budgets of less than $1 million reported higher levels of laboratory funding for patent prosecution.

One may observe from Fig. 4 that there is an increase in the proportion of patent prosecution funding from ORTAs as the size of the budget increases. In Fig. 4, ORTAs with budgets less than $1 million reported that the ORTA was the funding source 25% of the time; ORTAs with budgets between $1 million and $3 million reported funding from the ORTA 39% of the time; and ORTAs with budgets greater than $3 million reported the ORTA as the funding source 42% of the time. Likewise, there is a decrease in the funding of patent prosecution from the laboratories as the ORTA budget increases. ORTAs with budgets less than $1 million reported that the laboratory was the funding source 50% of the time; ORTAs with budgets between $1 million and $3 million reported funding from the laboratory 22% of the time; and ORTAs with budgets greater than $3 million reported the laboratory as the funding source 17% of the time. This suggests that ORTAs with larger budgets have more responsibility for funding patent prosecution.

In addition to asking respondents about the source of funds for patent filing, the survey asked if the ORTA uses internal counsel, external counsel, or a combination of the two to prosecute its patents. We describe this as the patent filing source for the ORTA. These differing sources of patent filing may, in theory, have different effects on the decision as to which patents are filed.

**Fig. 5:**
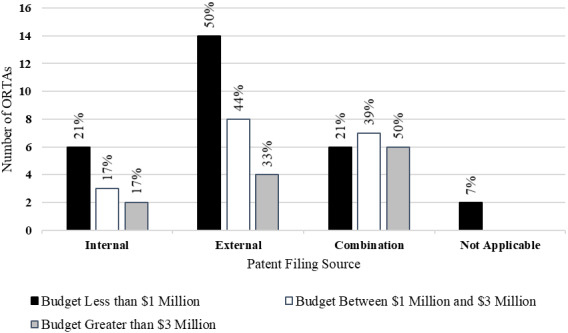
Patent filing source by budget category (*n* = 58).

Figure 5 shows the patent filing source for ORTAs. This question asked respondents about whether patents were prosecuted internally, externally, or as some combination of internal and external. ORTAs with budgets of less than $3 million reported higher rates of external patent filing than did ORTAs with budgets greater than $3 million.

Siegel *et al*. [19] found that increased expenditures on external counsel for intellectual property management resulted in a reduced number of licensing agreements but increases in licensing revenues. The authors suggested that external counsel was viewed as more aggressive in negotiation with potential licensees.

One may observe from Fig. 4 and Fig. 5 that ORTAs with smaller budgets utilized external funding and external counsel at higher rates than ORTAs with larger budgets. This likely reflects the ability to leverage larger pools of internal resources by ORTAs with larger budgets.

We next discuss the distribution of royalties to inventors. The proportion of royalties distributed to inventors, in theory, may influence their incentives and behaviors. In theory, higher royalty compensation may incentivize higher rates of invention disclosure. Further, to the extent that inventors play a role in the licensing of their inventions, higher royalty rates paid to inventors may incentivize them to more actively promote their inventions to potential licensees.

**Fig. 6:**
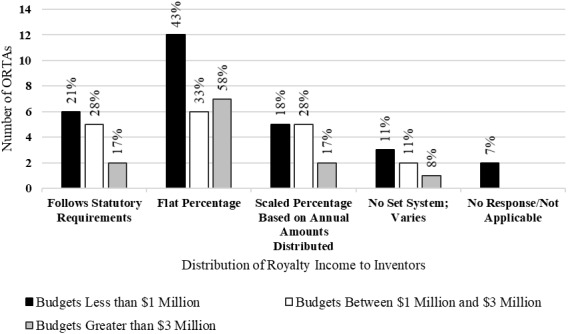
Distribution of royalty income to inventors by budget category (*n* = 58).

Figure 6 shows the distribution of royalty income to inventors. All ORTAs follow the statutory guidelines, but some exercise the option to provide additional incentives to inventors. For example, ORTAs may offer an increased percentage of the royalty income to inventors. ORTAs reported, with the greatest frequency, providing a flat percentage of licensing royalties to inventors. However, ORTAs with budgets of less than $3 million reported following statutory guidelines when providing royalties to inventors with a higher frequency than did ORTAs with budgets greater than $3 million. Royalty distribution differed across and within federal agencies. Further, in some cases, royalty distribution differed among laboratories with similar structures (i.e., GOGO/GOCO) within the same agency.

Thursby *et al*. [27] found that universities distributed on average approximately 40% of the royalties to inventors. This figure is significantly above the statutory minimum of 15% provided by federal ORTAs. Additionally, federal ORTAs, with some exceptions, statutorily cap royalties to inventors at $150,000. The authors make no mention of an upper bound on the royalties that universities award to inventors.

Following on from the distribution of royalties to inventors, we next discuss the distribution of remaining royalties. As with the distribution of royalties to inventors, the distribution of the remaining royalties may influence the incentives for various decision makers in the technology transfer process. Parties that receive a higher proportion of the remaining royalties may, in theory, be more incentivized to actively participate in the technology transfer process. One may also imagine that inventors who have altruistic feelings towards their own laboratories may internalize the benefits of the royalties that are distributed to said laboratories.

**Fig. 7:**
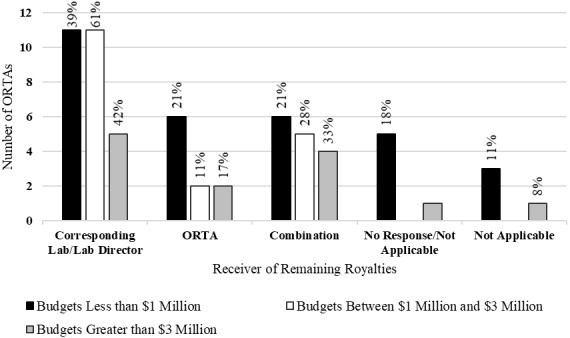
Receiver of remaining royalties by budget category (*n* = 58).

Figure 7 shows the receiver of remaining royalties after distribution of royalty income to inventors. The majority of all ORTAs distributed excess royalties to the corresponding laboratory/laboratory director. This was the most common response, but it did not reach a majority for ORTAs with budgets greater than $3 million. Relative to ORTAs with budgets less than $3 million, ORTAs with budgets greater than $3 million reported higher rates of retaining excess royalties in the ORTA or a combination of distribution between the laboratory and the ORTA.

Thursby *et al*. [27] found that universities split the royalties remaining after compensating the inventors as follows: 16% to the corresponding laboratory, 26% to administration, and 11% to the ORTA. Studies that examined the relationship between royalty payments to inventors and technology transfer output found that there were positive relationships between the royalty payments to inventors and the licensing revenues of the ORTA [27, 29–30]. Link and Siegel [30] also reported that universities with higher royalties paid to inventors were more efficient in their technology transfer activities.

This relationship between royalty shares and technology transfer has been observed in universities outside of the United States. Caledera and Debande [28] found a positive relationship with royalty shares allocated to inventors and the licensing revenues of Spanish universities. They did not, however, observe an increase in the number of licenses. Walter *et al.* [31] studied the incentives of researchers at German universities. They found that these researchers responded positively to financial and career-enhancing incentives. In particular, the authors found that researchers responded to larger shares of royalties distributed to the inventors and larger shares of royalties distributed to research groups.

While we are unable to observe the behaviors of inventors at governmental laboratories, if researchers at government laboratories could be expected to respond similarly to incentives as do researchers at universities, we would expect higher rates of invention disclosures where ORTAs give relatively larger shares of royalties to the inventors and the inventors’ corresponding laboratories.

The survey also asked questions about the role of the ORTA in activities that support technology transfer. Specifically, it asked questions about how the ORTA conducted market analysis for its technologies and the role the ORTA played in the CRADA approval process. The theoretical relationship between the ORTA’s role in these supporting activities and technology transfer outcomes is not readily apparent. It may be the case that more direct involvement by the ORTA could result in a more efficient process due to the institutional knowledge of the ORTA. Alternatively, more involvement by the ORTA could add an additional barrier and cause delays.

The survey asked whether ORTAs conducted market analysis of technologies internally (i.e., using ORTA or laboratory personnel), externally (i.e., hiring an external evaluator), or a combination of the two. The most common response of ORTAs across all budget categories was that they conducted market analyses of new inventions internally. This reported rate was higher for ORTAs with larger budgets and was a majority for ORTAs with budgets greater than $1 million. No ORTAs with budgets greater than $3 million reported conducting market analyses exclusively externally.

**Fig. 8:**
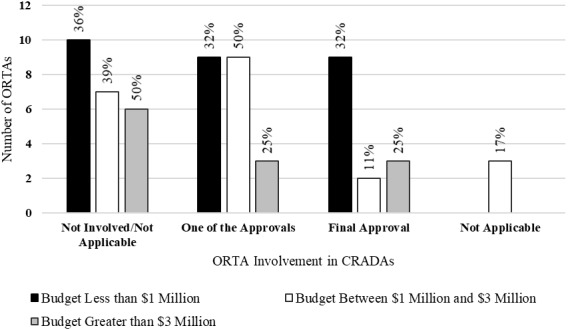
ORTA involvement in CRADAs by budget category (*n* = 58).

Figure 8 shows the role that ORTAs play in the approval of CRADAs. ORTAs with budgets less than $3 million played a slightly smaller role in the approval of CRADAs than ORTAs with budgets greater than $3 million. ORTAs with smaller budgets reported having approval authority at a rate of 50%. ORTAs with larger budgets reported having approval authority at a rate of 66%.

There has been a relatively limited treatment of CRADAs in the academic literature. Chen *et al*. [32] noted that much of this limited research consists of case studies examining specific CRADAs. They [32] examined the CRADA agreements at NIST, focusing on the external entities that chose to enter into NIST agreements. Among their findings, they noted that large businesses were more likely to engage in multiple CRADAs. Adams *et al*. [33] found that CRADAs generated greater numbers of industrial patents and industrial R&D than other forms of technology transfer. Jaffe and Lerner [34] studied technology transfer from U.S. Department of Energy laboratories and found, among other things, that the CRADA approval process was excessively burdensome. Rogers *et al*. [35] echoed this sentiment when they found that firms were critical of the time requirement and complexity of CRADA formation.

## 5 Conclusion

It has been a long-held understanding that the benefits of investments in science by the federal government are realized when the knowledge developed from these investments is transferred to society. The ORTAs at federal laboratories are charged with the task of facilitating this transfer. However, there has been a limited body of research on the operations of federal ORTAs compared to the literature on their university counterparts. This work provides a summary of responses to a survey of ORTAs at government laboratories and provides an overview of relevant peer-reviewed literature on the relationship between ORTA characteristics and technology transfer performance.

This paper provides an initial overview of the organization and operation of ORTAs. This work is descriptive in nature and presents some generalizations drawn from survey responses. These generalizations may serve as preliminary findings and a basis for future research on government laboratories. In summary, first, ORTAs with larger budgets generally had higher levels of human capital resources (i.e., FTEs) and were more likely to conduct technology transfer activities using internal resources (e.g., internal counsel). Second, ORTAs responded that they employed several different compensation schemes for rewarding their inventors. This information may be leveraged in future research to examine the effects that incentives have on invention disclosures at government laboratories. These insights are important because these characteristics have been observed to be related to technology transfer performance at ORTAs housed within universities.

We urge the reader to consider several limitations of the analysis of the survey information when interpreting results. First, the information presented is from 58 ORTAs, whereas there are hundreds of federal laboratories. It is possible that these data may suffer from selection bias (e.g., larger ORTAs may be more likely to respond than smaller ORTAs). As such, we caution against using this initial analysis to generalize about the entire population. Second, this work only describes the characteristics of ORTAs at federal laboratories and the relevant research on university ORTAs, it does not include information on the technology transfer outcomes, and only a few of the potential mechanisms for technology transfer described by Hourihan [7] were considered.

Additionally, while one may draw some lessons from the peer-reviewed literature on university ORTAs, Lafuente and Berbegal-Mirabent [3] cautioned that “knowledge of their internal resource usage can help [ORTA]s be more efficient. Conversely, changing your operations based on others may lead to inefficiencies and ‘poor productivity results.’” This suggestion for caution is enhanced by the potential differences in missions between universities and federal laboratories, which likely influence the organization and operation of their respective ORTAs.

Further, Berbegal-Mirabent et al. [36] found that factors external to the organization (e.g., regional factors) may influence the technology transfer efficiency of an ORTA. Further, they observed that the optimal technology transfer policy may vary based on these external factors. This suggests that the characteristics of ORTA organization and operation may have adapted over time in response to the needs of the individual laboratories and agencies.

We suggest two areas for future research following this initial look at federal ORTAs. In the first area, researchers may measure the relationship between technology transfer activities (i.e., patents, licenses, licensing revenues) and data on federal ORTAs’ organizational and operational characteristics to produce analyses comparable to research on university ORTAs. One topic in this research is the estimation of the elasticity of technologies transferred with respect to additional ORTA funding at federal laboratories. In the second area, researchers may follow the suggestions of Balven *et al*. [37] and consider the microprocesses that influence inventors at federal laboratories to engage in technology transfer activities. One topic in this research area is an assessment of the relationship between alternative royalty schemes and the motivation of inventors to engage in technology transfer activities at federal laboratories. Both research areas would benefit from enhanced data collection efforts.
